# Linking maternal blood pressure with fetal cerebral haemodynamics and cortical growth in congenital heart disease

**DOI:** 10.1016/j.ebiom.2026.106367

**Published:** 2026-07-06

**Authors:** Siân Wilson, Seungyoon Jeong, Louise Wilkins-Haug, P. Ellen Grant, Caitlin K. Rollins, Sarah U. Morton, Kiho Im

**Affiliations:** a Fetal-Neonatal Neuroimaging & Developmental Science Centre, Boston Children’s Hospital, Boston, MA, 02115, USA; b Division of Newborn Medicine, Boston Children's Hospital, Boston, MA, 02115, USA; c Department of Pediatrics, Harvard Medical School, Boston, MA, 02115, USA; d Division of Maternal Fetal Medicine, Brigham and Women’s Hospital, Boston, MA, 02115, USA; e Department of Radiology, Boston Children’s Hospital, Boston, MA, 02115, USA; f Department of Neurology, Boston Children’s Hospital and Harvard Medical School, Boston, MA, 02115, USA; g Department of Obstetrics, Gynecology & Reproductive Biology, Harvard Medical School, Boston, MA, 02115, USA

**Keywords:** Blood pressure, Brain, Congenital heart disease, Foetal, Maternal, MRI, Doppler

## Abstract

**Background:**

Children with congenital heart disease (CHD) experience neurodevelopmental challenges, arising in part from altered brain maturation beginning in utero. There are currently no prenatal interventions to mitigate this risk. Foetal CHD is associated with alterations in maternal blood pressure (BP), for which therapies are available, so we investigated associations between maternal BP, foetal cerebral haemodynamics, and brain growth in foetuses with and without CHD.

**Methods:**

Our single-centre retrospective cohort study first analysed maternal BP during pregnancy stratified by foetal CHD (n = 494), other foetal anomalies (n = 769), or no foetal anomalies (n = 111). We then performed a prospective study linking maternal BP with foetal brain MRI and cerebral and umbilical Doppler measures in pregnancies with foetal CHD (n = 97 MRI; 121 Doppler) and those with no foetal anomalies (n = 111 MRI; 86 Doppler).

**Findings:**

Mothers carrying a foetus with CHD showed a distinct BP profile compared with unaffected controls and non-CHD foetal anomaly pregnancies. In CHD pregnancies, but not in controls, lower maternal diastolic BP was associated with lower cerebrovascular resistance, lower cerebroplacental flow ratio, and an attenuated reduction in foetal cortical surface area in sensorimotor, frontal and temporal cortical regions.

**Interpretation:**

Lower maternal diastolic BP may reflect adaptive maternal–foetal circulatory coupling that enhances foetal cerebral perfusion and mitigates cortical growth impairment in CHD. We observe a link between maternal haemodynamics and foetal brain maturation in CHD, warranting further exploration in interventional studies.

**Funding:**

Supported by the 10.13039/100000065NINDS (R01NS114087, K23NS101120), 10.13039/100000070NIBIB (R01EB031170), 10.13039/100000050NHLBI (K08HL157653), AAN Clinical Research Training Fellowship, BBRF Young Investigator Awards, and the Farb Family Fund.

**Research in context**

**Evidence before this study**

We searched the PubMed database in January 2025 using terms related to maternal–foetal health, congenital heart disease (CHD), and foetal/neonatal brain imaging (for example: “MRI”, “foetal”, “neonatal”, “prenatal”, “postnatal”, “congenital heart disease”, “hypertension”, “blood pressure”, “stress”, “Doppler”). Prior work links qualitative assessment of maternal stress, diabetes, and hypertensive disorders to adverse brain development in offspring in the general population, while several other studies have described altered cerebral oxygenation and cerebrovascular reactivity in foetuses with CHD. However, quantitative physiological markers of maternal stress such as maternal blood pressure or cardiac output have not been linked to foetal cerebral haemodynamics or brain growth in foetuses with CHD.

**Added value of this study**

Integrating maternal blood pressure, umbilical and foetal cerebral Doppler, and foetal MRI, we examine the relationship between maternal haemodynamics, foetal-placental blood flow, and foetal cortical growth. We show that isolated low diastolic blood pressure is enriched in pregnancies with foetal CHD, compared to pregnancies with non-CHD anomalies or no anomalies. We then associate lower maternal diastolic blood pressure with reduced resistance to blood flow to the foetal brain, and less impaired cortical areal growth in foetuses with CHD. We accounted for medication use, placental pathology, estimated foetal weight and other potential confounders, and contrasted findings in CHD vs. control pregnancies, providing multimodal evidence that variation in maternal haemodynamics could be an adaptive compensation that leads to resilience in foetal cerebral perfusion and brain development in CHD.

**Implications of all the available evidence**

These findings highlight maternal cardiovascular physiology as a potentially important, and previously underappreciated, modifiable factor linked to foetal brain growth and resilience in CHD. These observations invite future interventional studies to investigate whether maternal blood pressure could be monitored or modulated to support foetal brain health in pregnancies affected by CHD. Greater understanding of the factors that regulate this potentially adaptive physiology may improve individualised risk assessment during pregnancy and inform the development of prenatal strategies that enhance neurodevelopmental resilience.

## Introduction

Congenital heart disease (CHD) is the most common major congenital anomaly, affecting 1% of live births.^1^^,^^2^ Despite advances in surgical care, it is a leading cause of paediatric morbidity.^3^ Many children with CHD experience impaired neurodevelopment, and increasing evidence suggests this health risk may have prenatal origins.^4^ Observational brain magnetic resonance imaging (MRI) studies have shown structural abnormalities in the foetal brain are linked to poorer cognitive, motor, and language outcomes in toddlerhood.^5^^,^^6^ These abnormalities include reduced overall brain size, the volume of transient foetal compartments such as the subplate^5^ and cortical grey matter volume.^7^, ^8^, ^9^, ^10^ In particular, recent work highlighted that deviations from normative cortical growth trajectories are associated with worse neurodevelopmental outcomes at two years,^6^ emphasising in utero cortical growth and maturation, specifically reduced regional cortical areal expansion, as potential structural correlates of future neurodevelopmental deficits.^6^ Notably, substantial variation in cortical surface area was observed within the CHD cohort that was not attributable to CHD diagnosis alone,^6^ underscoring the need to identify other antecedent factors contributing to this heterogeneity. Thus far, the mechanisms linked to disrupted foetal brain maturation include genetic factors, reduced cerebral substrate delivery, placental dysfunction, and maternal stress.^4^

The maternal–foetal environment is increasingly recognised as an understudied but important proponent of risk and resilience in brain development. Although questionnaire-based measures of maternal stress have previously been linked to foetal brain structure differences in pregnancies with foetal CHD,^11^ physiological markers of maternal cardiovascular tone are understudied mediators of foetal brain maturation.^11^ Furthermore, preexisting maternal conditions such as hypertension, diabetes, and obesity are associated with an increased risk of a foetus with CHD, and pregnancy-related hypertensive disorders occur more commonly during pregnancy in individuals carrying a foetus with CHD.^12^, ^13^, ^14^, ^15^, ^16^, ^17^ Associations between these maternal or gestational hypertensive conditions and adverse neurodevelopmental outcome in offspring have also been observed,^17^^,^^18^ suggesting that maternal haemodynamics may play an important, unexplored role in shaping foetal brain development. For example, maternal–foetal circulatory coupling has been observed in CHD cohorts, including foetal autoregulatory responses to acute maternal hyperoxia.^18^^,^^19^ Maternal BP, a clinical marker of maternal cardiovascular function, is associated with foetal cardiac growth^20^ and cerebral perfusion in growth-restricted pregnancies^21^^,^^22^ but whether it influences foetal brain development in the context of CHD is not known. Given this evidence, we hypothesised that maternal haemodynamics, specifically blood pressure (BP), may be an important, uncharacterised mediator of foetal brain development in CHD.

In this study, we first explored whether the range of maternal BP values recorded were different in pregnancies with foetal CHD compared to pregnancies with other foetal anomalies, or no foetal anomalies. We then performed a secondary analysis of the subset of participants who participated in a prospective study that obtained foetal brain MRI and foetal/placental Doppler ultrasound measures to investigate the relationship between maternal BP during pregnancy, Doppler-derived measures of foetal cerebral arterial perfusion, and foetal cortical expansion assessed with brain MRI. To distinguish maternal haemodynamic effects from placental dysfunction, we also examined whether there was an effect of placental health, measured using standardised placental histopathology classifications. By integrating multimodal maternal, foetal, and placental measures across a large cohort, we identify links between maternal and foetal markers, informing potential avenues for future work on prenatal interventions to support resilience in foetal brain development.

## Methods

### Ethics

Ethical approval was granted by the Boston Children's Hospital Institutional Review Board for both the prospective (IRB approval number: IRB-P00008836) and retrospective (IRB-P00048815) components of this study. For the prospective recruitment of participants, written, informed consent was obtained from mothers prior to participation.

### Retrospective maternal BP cohort

**Fig. 1:**
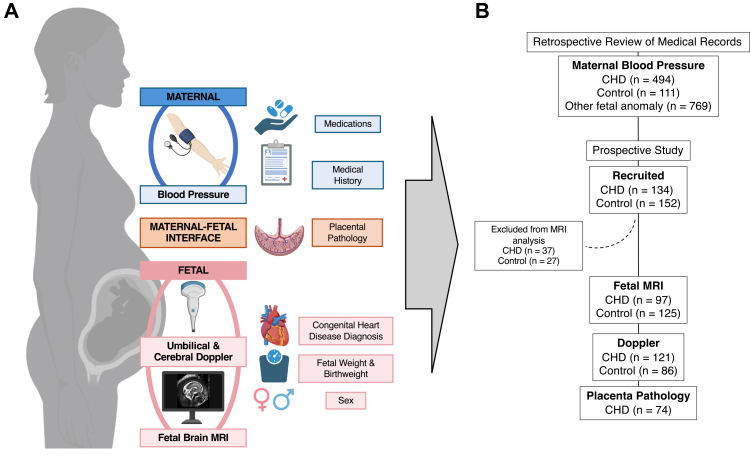
**Schematic of multimodal data collected to study maternal-foetal physiology in CHD.** (A) Primary outcome measures are highlighted in circles (maternal BP, umbilical and cerebral doppler, foetal brain MRI), and covariates/mediators accounted for listed on the right-hand side. **(B)** Study diagram of participants and data types collected in the retrospective maternal BP cohort and the prospectively enrolled MRI/Doppler subset; maternal BP values used in multimodal analyses were derived from the retrospective clinical dataset.

We conducted a retrospective assessment in May 2025 of maternal BP (systolic and diastolic) measures by extracting all blood pressure measurements obtained in the antenatal clinic at Boston Children’s Hospital from electronic medical records. We determined foetal diagnoses using the prospectively-maintained database of patients undergoing evaluation at the clinic (Fig. 1). The cohort included pregnancies with foetal CHD, a no-foetal anomaly group and an additional control subgroup, ‘non-CHD foetal anomaly’ to control for the potential confounding effects of stress in high-risk pregnancy, and other unmeasured external measurement factors. Foetal gestational age (GA) at measurement was calculated by backdating from birthdate and GA at birth. When multiple BP readings existed, the value closest to the date of the foetal brain MRI was selected. To account for the fluctuation in BP across pregnancy,^23^ raw BP values were converted to Z-scores based on a published reference (n = 36,000 pregnancies)^23^ using natural cubic spline interpolation.

### Prospective study: foetal brain MRI, cerebral and umbilical Doppler

A subset of the retrospective maternal BP cohort was enrolled in a single-centre prospective cohort study at Boston Children’s Hospital between 2014 and 2024, in which foetal brain MRI and Doppler ultrasound data were collected.^24^ Inclusion criteria included isolated moderate to severe foetal CHD^25^ or no foetal anomaly (unaffected controls), maternal age 18–45 years, singleton pregnancy. Exclusion criteria were maternal CHD, foetal genetic diagnosis, congenital infection, mild foetal CHD,^25^ or extracardiac anomalies or major brain malformations.

### Foetal MRI acquisition and processing

As described previously,^5^^,^^6^^,^^24^ foetal brain MRI was acquired between 20 and 39 weeks’ gestation on a Siemens 3T scanner using multi-planar repeated T2-weighted HASTE sequences (TE = 100–120 ms, TR = 1400–2000 ms, slice-thickness = 2–3 mm, in-plane resolution = 1 mm). Our in-house pipeline^6^^,^^26^^,^^27^ was used for image processing, including motion correction, slice-to-volume 3D reconstruction, brain extraction, and cortical plate segmentation. Cortical surfaces were reconstructed from the inner cortical plate boundary, registered to a 31 gestational week template and parcellated into 30 cortical regions per hemisphere.^26^^,^^28^ For each region, we calculated surface area then used normative modelling to obtain GA–adjusted z-scores representing individualised measures of cortical maturation, as described previously.^6^

### Foetal echocardiography and Doppler ultrasound

Foetal cerebral and umbilical Doppler measurements were taken by foetal sonographers in accordance with standard institutional clinical practice. Pulsed-wave Doppler was used to measure blood flow velocities in the middle cerebral artery (MCA) and umbilical artery (UA). Peak systolic velocity (PSV), end-diastolic velocity (EDV), and time-averaged mean velocity (TAMX) were recorded, and used to calculate the pulsatility index (PI) for each vessel with Equation (1).

1.PI=(PSV−EDV)/TAMX

A lower pulsatility index reflects reduced pulsatility with greater diastolic flow, indicating lower cerebrovascular resistance with higher, more continuous cerebral perfusion.

The cerebroplacental ratio (CPR) was calculated using Equation (3).

2.CPR=(MCAPI)/(UAPI)

Cerebroplacental ratio represents the balance of cerebral to placental vascular resistance and is used as way to assess foetal haemodynamics.^29^ Cerebroplacental ratio <1 indicates that the cerebrovascular resistance is lower than the placental resistance and is thought to reflect foetal cerebral redistribution or ‘brain sparing’ physiology.^30^ We computed z scores for middle cerebral artery pulsatility index, umbilical artery pulsatility index, and cerebroplacental ratio relative to GA using published reference data.^31^ Estimated foetal weight was calculated at each ultrasound using the Hadlock formula.^32^

### Medical history, placental pathology and birth information

Participants recruited to the prospective study provided body weight estimate and answered a questionnaire about medical history and medication usage (Supplementary Tables S1 and S2). Pregnancies were followed up and birthweight and placentas were collected at delivery. Histopathologic analysis was performed according to the Amsterdam criteria,^33^ with lesions classified as maternal vascular malperfusion, foetal vascular malperfusion, delayed villous maturation, or inflammatory lesions (Supplementary Table S7).

### Statistics

No formal a priori sample size calculation was performed, as this study comprised a retrospective analysis and a secondary analysis of an existing prospective cohort. All statistical analyses were performed in R version 2023.12.1 + 402 (R Foundation for Statistical Computing). A chi-square test was used to compare the distribution of patients between BP categories; and non-parametric t-test to compare BP z-scores between CHD and controls. To test for bimodality in the distribution of BP z-scores we used Hartigan’s dip test.

In a secondary analysis, we evaluated associations between maternal BP (systolic/diastolic) and foetal characteristics (brain growth, Doppler-derived estimates of foetal flow) using linear mixed-effects regression models. The prospective MRI/Doppler cohort was a subset of the larger retrospective maternal BP cohort, and for these participants the maternal BP values used in linked analyses were extracted from the retrospective clinical record dataset. For brain growth, foetal sex, GA at measurement, CHD subtype (single vs. two ventricle physiology), maternal body mass index (BMI), maternal age, and a random factor (1/Subject) were included as covariates to account for repeated measures (2 MRI timepoints). Non-significant sociodemographic covariates were removed from the final model equations (e.g., parity, gravidity, maternal education).

For Doppler measures, we also fit linear mixed effects regression models, including foetal sex, GA, CHD subtype, maternal age, maternal BMI, foetal head circumference and estimated foetal weight as covariates. Head circumference and estimated foetal weight were included to control for foetal size–related differences in flow velocity and to disentangle the direct haemodynamic effects of maternal blood pressure from those secondary to foetal growth variation. Statistical significance was defined as *q* < 0.05, using false discovery rate (FDR) to correct for multiple comparisons.

### Role of funders

The funders did not play any role in the study design, data collection, data analyses, interpretation, or writing of the manuscript.

## Results

### Participants

In the cohort with maternal BP measurements from the antenatal clinic (Fig. 1), we considered three groups: (1) foetuses with CHD (n = 494), (2) foetuses with other congenital anomalies (n = 796), and (3) foetuses with no congenital anomalies (n = 111). There was no difference in mean [SD] maternal age in years between groups: CHD, 31.4 [5.31]; other congenital anomalies, 32.1 [5.48]; and no congenital anomalies, 33.4 [5.22] (F(2, 1398) = 2.55, p = 0.80). The subset of participants with Doppler and MRI data were in two groups: (1) foetuses with moderate-to-severe CHD and (2) controls with no foetal anomaly (Fig. 1). After quality control for motion and distortion-corrupted data, foetal brain MRI data were available for 222 participants (CHD = 97, Control = 125), Doppler ultrasound data for 207 (CHD = 121, Control = 86), and placental pathology for 74 (all CHD; Fig. 1). The distribution of participants between BMI categories did not differ between groups, however mean (SD) pre-pregnancy BMI was higher in the CHD group (CHD vs. Control, 26.6 [5.16] vs. 24.5 [5.06]; Welch t = −2.81, 95% CI (−3.51, −0.61), p < 0.01), while maternal age in years did not differ (31.9 [4.54] vs. 33.4 [4.12]; Welch t = −2.39, 95% CI (−1.84, 0.87), p = 0.48; Supplementary Table S1). Only 3% of mothers reported antihypertensive use, and there was generally low medication use in both groups, so we lacked statistical power to test if these exposures were relevant to our outcomes (Supplementary Table S2).

### Distinct and bimodal maternal diastolic blood pressure profile in pregnancies with foetal CHD

**Fig. 2:**
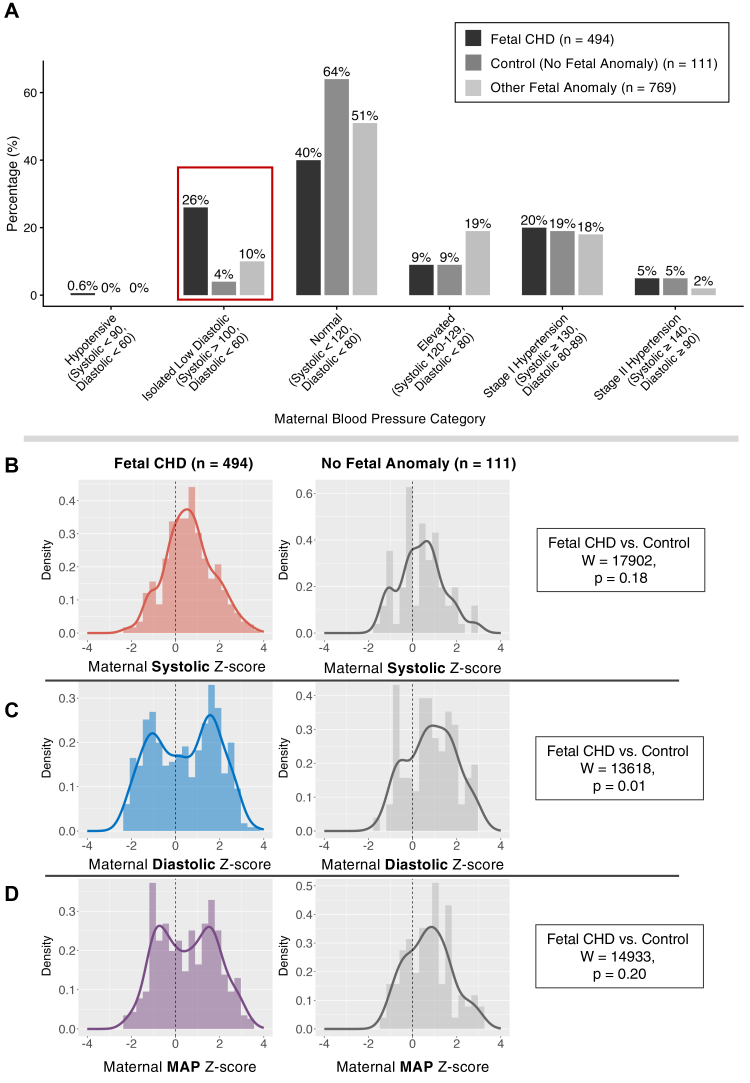
**Mothers carrying a foetus with CHD show greater incidence of isolated low diastolic blood pressure during pregnancy. (A)** Categorised blood pressure (BP) values for CHD (n = 494), Control (No foetal anomaly) (n = 111) and non-CHD foetal anomaly (n = 769) pregnancies. Stacked bar plot shows the percentage of participants in each BP category (Hypotensive, Isolated Low Diastolic, Normal, Elevated, Stage I Hypertension, Stage II Hypertension). Global chi-square testing showed a difference in the distribution between the three participant groups (χ^2^(df = 10) = 108.77, p < 0.01). Pairwise comparisons showed differences between all groups; CHD vs. No Foetal Anomaly (Controls) (χ^2^(df = 5) = 32.22, p < 0.01), Control vs. Non-CHD Foetal Anomaly (χ^2^(df = 5) = 15.42, p = <0.01), and CHD vs. Non-CHD Foetal Anomaly (χ^2^(df = 5) = 85.48, p < 0.01). **(B–D)** Histograms showing the distribution of maternal BP z-scores with Foetal CHD (Left) and Controls (Right) pregnancies. **(B)** Red = Systolic z-scores, **(C)** Blue = Diastolic z-scores and **(D)** Purple = Mean Arterial Pressure (MAP) z-scores. Dashed vertical lines represent the Loerup et al., reference mean (z = 0) used to derive the z-scores. Statistical testing indicated bimodal distribution of Diastolic z-scores (Hartigan’s dip test: D = 0.05, p < 0.01) and MAP z-scores (D = 0.05, p = 0.03) in CHD pregnancies. Wilcoxon rank-sum testing demonstrated that Diastolic and MAP z-scores were different between CHD and Control pregnancies (W = 10,989, p-value <0.01; W = 11,702, p-value = 0.02), whereas systolic did not differ between groups (W = 13,940, p-value = 0.78).

**Fig. 3:**
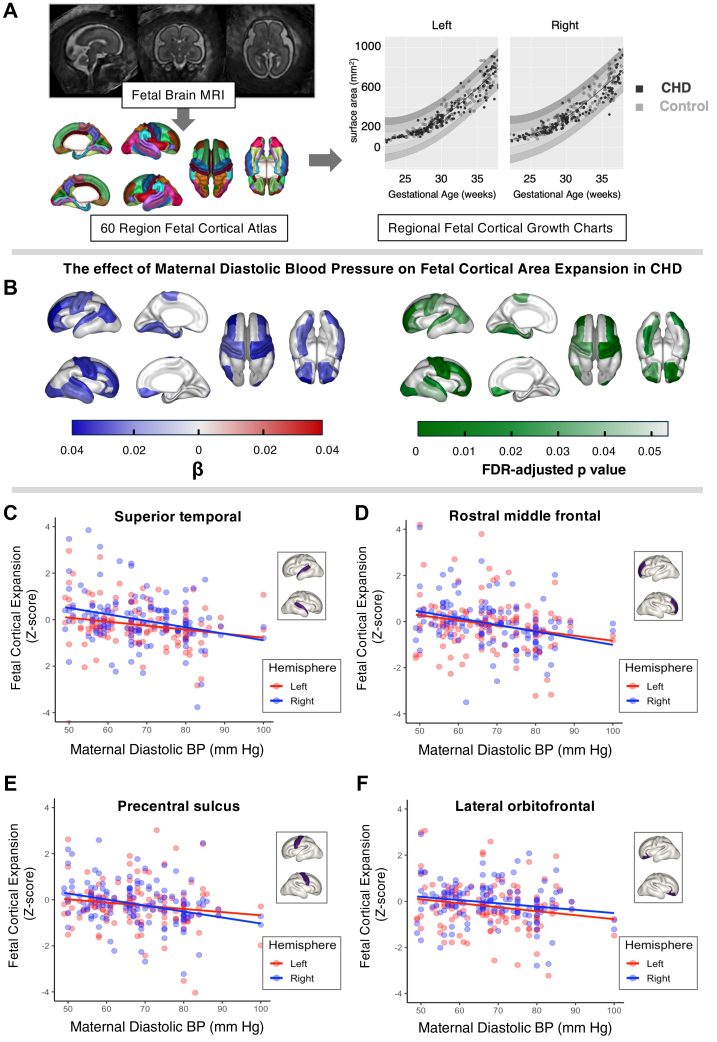
**The effect of maternal diastolic blood pressure on foetal cortical growth in CHD. (A)** Simplified foetal MRI methods schematic illustrating how cortical growth measures (z-scores) are derived from foetal brain MRI. Foetal MRI volumes are processed and registered to a foetal surface atlas, then cortical metrics are calculated for each region of the atlas and normative modelling is used to chart their maturation across age, growth charts (right) show the model mean and ±1, ±2, and ±3 standard deviations, with foetuses with CHD overlaid in black (Adapted with permission from Wilson et al., 2025) **(B)** In foetuses with CHD, Maternal Diastolic BP is a predictor of foetal cortical expansion in 27 regions (labelled with effect size and corresponding p-values). **(C–F)** Exemplary plots of the relationship between surface area Z-score and maternal diastolic BP for cortical regions where maternal BP had a effect in both left and right hemispheres. Abbreviations: BP, blood pressure; CHD, congenital heart disease; FDR, false discovery rate; MRI, magnetic resonance imaging.

Mothers carrying a foetus with CHD showed a distinct BP profile compared with both controls (no foetal anomaly) and non-CHD foetal anomaly pregnancies (Fig. 2; χ^2^ = 108.77, df = 10, p < 0.01), with CHD pregnancies showing a higher incidence of isolated low diastolic BP (26%) compared to 4% in Controls, and 10% in Non-CHD Foetal Anomalies (Fig. 3A). This skewed distribution was persistent when stratified by trimester (Supplementary Fig. S1A and B). Pairwise comparisons confirmed differences between CHD vs. controls (χ^2^ = 32.22, df = 5, p = 0.01), CHD vs. Other foetal anomalies (χ^2^ = 85.48, df = 5, p < 0.01), and controls vs. Other foetal anomalies (χ^2^ = 15.42, df = 5, p < 0.01). For CHD and control pregnancies, we used foetal GA at the time of BP measurement to calculate z-scores for maternal systolic, diastolic and mean arterial pressure relative to GA (Fig. 2B–D), based on a reference average from 36,000 uncomplicated pregnancies^23^ (Supplementary Fig. S1C). In CHD pregnancies, Diastolic BP Z-scores had a bimodal distribution (Hartigan’s dip test: D = 0.04, p < 0.01; Fig. 2C) as did mean arterial pressure (D = 0.03, p = 0.03; Fig. 2D). There was a significant difference in diastolic z-scores between CHD and control pregnancies (W = 13,618, p = 0.01), whereas systolic and mean arterial pressure did not reach statistical significance when comparing groups (W = 17,902, p = 0.18; W = 14,933, p = 0.20; Supplementary Table S3).

### Maternal diastolic BP is associated with cortical expansion in foetuses with CHD

We used individualised measures of regional foetal cortical growth published previously,^6^ focusing on cortical surface area z-scores, as this feature of cortical morphometry differentiated foetuses with CHD from unaffected controls in our previous study, and was associated with neurodevelopmental outcome (Fig. 3A). Among foetuses with CHD, maternal diastolic BP was negatively associated with cortical surface area z-scores in 27 out of 60 cortical regions, after FDR correction for multiple comparisons (Fig. 3B, Supplementary Table S4). Representative bilateral regions included the superior temporal (β = −0.025 z-score/mmHg, 95% CI −0.041 to −0.009, p = 0.022; β = −0.030 z-score/mmHg, 95% CI −0.050 to −0.010, p = 0.014), rostral middle frontal (β = −0.038 z-score/mmHg, 95% CI −0.058 to −0.018, p = 0.007; β = −0.032 z-score/mmHg, 95% CI −0.050 to −0.014, p = 0.005), precentral (β = −0.020 z-score/mmHg, 95% CI −0.036 to −0.004, p = 0.028; β = −0.031 z-score/mmHg, 95% CI −0.047 to −0.015, p = 0.005), postcentral (β = −0.022 z-score/mmHg, 95% CI −0.040 to −0.004, p = 0.044; β = −0.033 z-score/mmHg, 95% CI −0.051 to −0.015, p = 0.005), and lateral orbitofrontal cortices (β = −0.023 z-score/mmHg, 95% CI −0.041 to −0.005, p = 0.028; β = −0.025 z-score/mmHg, 95% CI −0.039 to −0.011, p = 0.006) (Fig. 3C–F). We found no significant effect of systolic BP on cortical surface area among foetuses with CHD (Supplementary Table S4), or for either BP measurement in controls (Supplementary Table S5).

### Maternal diastolic BP is associated with cerebrovascular resistance but not umbilical artery resistance in foetal CHD

**Fig. 4:**
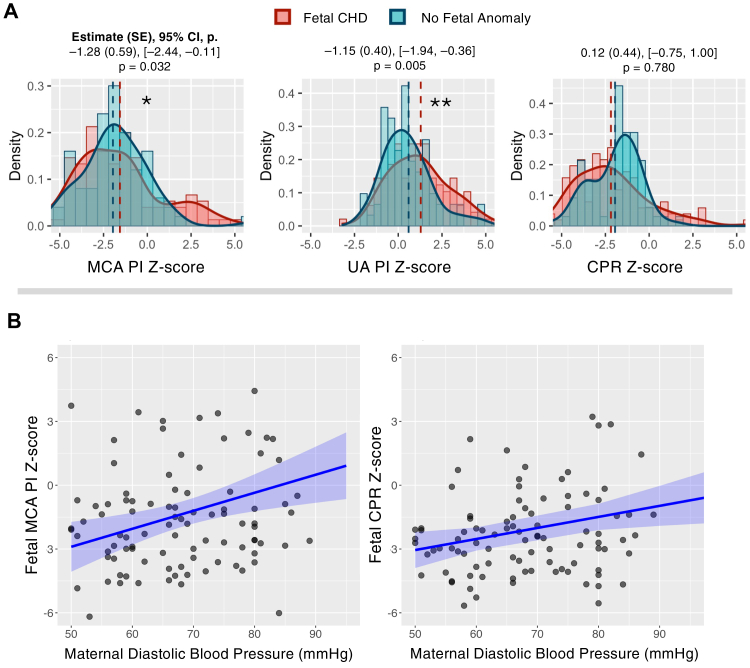
**Maternal diastolic BP is associated with cerebral blood flow in foetuses with CHD. (A)** Comparing the distribution of Z-scores of Foetal Doppler measurements: Pulsatility Index (PI) Z-scores in foetuses with CHD (red) vs. no foetal anomaly (blue), in the Middle Cerebral Artery (MCA), Umbilical Artery (UA) and Cerebroplacental Ratio (CPR). Statistics reflect the group difference (CHD – No Foetal Anomaly) in z-score for each Doppler measure, adjusted for foetal sex, GA, single ventricle physiology, maternal age, maternal BMI, foetal head circumference and estimated foetal weight. **(B)** (Left) The relationship between maternal diastolic blood pressure (mmHg) and foetal MCA PI. (Right) The relationship between maternal diastolic blood pressure (mmHg) and foetal CPR ratio. Each point represents an individual observation, and the solid blue line shows the adjusted effect of diastolic BP on MCA PI from a multivariate linear regression model (holding continuous covariates at their mean; GA, single ventricle physiology, maternal age, maternal BMI, foetal head circumference) with the shaded blue area representing the 95% confidence interval.

To explore a potential mechanism by which maternal BP impacts foetal brain development in CHD, we investigated the association between maternal BP and umbilical arterial or cerebral doppler indices. Compared to foetuses without CHD, foetuses with CHD had lower middle cerebral artery pulsatility index Z-scores (mean difference, −1.28 [SE, 0.59]; 95% CI, −2.44 to −0.11; p = 0.03) and umbilical artery pulsatility index Z-scores (−1.15 [SE, 0.40]; 95% CI, −1.94 to −0.36; p = 0.005), whereas we did not observe a significant difference in cerebroplacental ratio Z-scores between groups (0.12 [SE, 0.44]; 95% CI, −0.75 to 1.00; p = 0.78) (Fig. 4A). In the CHD group, lower maternal diastolic BP was associated with lower middle cerebral artery pulsatility index (β = 0.08 z-score/mmHg; 95% C.I, 0.03–0.139; p < 0.01) and cerebroplacental ratio (β = 0.05 z-score/mmHg; 95% C.I, 0.013–0.091; p = 0.01) after adjustment for single ventricle physiology, GA, foetal sex, head circumference z-score, and maternal age, consistent with cerebral vasodilation and brain-sparing physiology, while no statistically significant associations were observed for umbilical artery indices, including umbilical artery pulsatility index (β = −0.005 z-score/mmHg, SE = 0.02, 95% CI -0.040 to 0.030, p = 0.782) and umbilical artery resistive index (β = −0.02 z-score/mmHg, SE = 0.02, 95% CI -0.047 to 0.017, p = 0.345; Fig. 4B and C, Supplementary Table S6).

### Maternal BP is not associated with placental pathology, umbilical artery flow, or foetal growth

Placenta pathology that reflected vascular malperfusion or inflammation was evaluated in pregnancies with foetal CHD (n = 74). Maternal vascular malperfusion was present in 23 placentas (31%), foetal vascular malperfusion in 8 (11%) and placental inflammation in 24 (32%; Supplementary Table S7). No associations were observed between maternal BP and the presence of placental pathology features (Supplementary Fig. S2), or between maternal BP and estimated foetal weight (Supplementary Table S8).

## Discussion

In this cohort study of pregnancies affected by moderate to severe foetal CHD, we observed lower maternal diastolic BP being associated with an attenuated reduction in cortical surface area expansion in the foetal brain, linking lower maternal diastolic to improved cortical growth and maturation in foetuses with CHD. These associations were specific to pregnancies with foetal CHD and were accompanied by reduced cerebrovascular resistance without change in umbilical artery flow or placental pathology. These observations suggest a previously uncharacterised link between maternal cardiovascular physiology and ‘resilience’ in the maturation of foetal cortical expansion in CHD. While causality cannot be inferred, these findings emphasise the importance of studying the dynamic interplay between maternal and foetal systems to explain the variability in brain development in foetal CHD.

In a retrospective analysis of over 1200 blood pressure measurements from antenatal clinic visits, we identified a unique maternal BP phenotype, isolated low diastolic BP, as being disproportionately common in pregnancies with foetal CHD compared with other foetal anomalies or unaffected foetuses. Isolated low diastolic BP was recorded in 26% of pregnancies with foetal CHD, compared to 4% in controls and 10% in the ‘other foetal anomaly’ group, and was persistent when measurements were stratified by trimester, and z-scored relative to GA. In this study we report a high incidence of isolated low diastolic BP, introducing a new physiological observation for clinical consideration in pregnancies with a foetal CHD diagnosis. The distinctness of the isolated low diastolic phenotype to CHD pregnancies compared to ‘other foetal anomaly’ controls suggests this observation is not attributable to nonspecific features of a high-risk pregnancy or measurement variability. All BP measurements were taken in the same clinical setting, somewhat minimising external confounding influences on our analysis. While there is an established association between maternal hypertensive disorders of pregnancy and foetal CHD,^34^ we instead found a non-hypertensive trait being enriched in pregnancies with foetal CHD. We hypothesise that this phenotype may represent a maternal adaptation emerging in response to altered foetal circulatory demands in CHD, though further longitudinal data and ambulatory monitoring is required to test this hypothesis.

The mechanisms by which maternal cardiac output may influence foetal brain and body growth in CHD remain unclear, and there is an absence of literature in this area. Based on our observations, we hypothesise that lower maternal diastolic BP could reflect a compensatory maternal–foetal circulatory adaptation to promote foetal brain perfusion in the context of CHD, thereby supporting otherwise compromised maturational processes (such as cortical expansion).^6^ Lower maternal diastolic BP, but not systolic BP, was associated with reduced middle cerebral artery pulsatility index and cerebroplacental ratio, suggesting a pattern consistent with increased foetal cerebral blood flow relative to systemic circulation.^35^^,^^36^ On the other hand, estimated foetal weight and birthweight were not associated with maternal BP or Doppler measures, supporting the interpretation that this circulatory adjustment may preferentially sustain brain development rather than general somatic growth.

Finally, supporting a role in brain maturational resilience, lower maternal diastolic BP was associated with less impaired cortical surface area expansion across pre- and post–central, frontal and temporal cortical regions, linking maternal cardiovascular physiology to foetal brain growth. The lack of evidence that placental pathology and umbilical artery flow were impacted by maternal BP suggests our observations reflect maternal haemodynamic variation rather than placental insufficiency. However, our placental measures reflect placental health after delivery, and we did not assess placental biomarkers, such as the sFlt-1/PlGF (Soluble fms-Like Tyrosine Kinase-1/Placental Growth Factor) ratio, which are better markers of placental insufficiency and offer more predictive, temporally relevant insights.^37^^,^^38^ Further, there could be differences in placental vascular compliance that are not captured by current histologic assessments of placental pathology.

In support of our results, previous work showed cerebrovascular reactivity in third-trimester foetuses with CHD,^18^^,^^35^^,^^39^^,^^40^ measured in response to administered maternal hyperoxia.^40^ Greater cerebrovascular reactivity was associated with larger foetal brain volume and less postnatal white matter injury.^39^ Conversely, a lack of response to maternal hyperoxia has been associated with worse neurological outcomes.^41^ To our knowledge, no prior study has investigated maternal BP in relation to the foetal brain in CHD. However, in a cohort of over 8000 pregnancies, lower third-trimester BP was associated with larger foetal head circumference, with the strongest effect observed for diastolic pressure at more advanced GA.^42^ This convergence of evidence suggests that maternal circulatory tone may influence foetal cerebral perfusion and, importantly, represents a clinically actionable pathway given the availability of established therapies for blood pressure modulation during pregnancy. We propose that in foetuses with moderate to severe CHD, maternal cardiovascular adaptation may serve as a compensatory mechanism to maintain foetal cerebral perfusion and support resilience in foetal brain maturation.

To build on the findings reported in this study, there are several avenues for further investigation. This is a single centre study that used retrospective data and a secondary analysis of a prospective study focused on complex, severe types of CHD without specific subgroup analysis by CHD type, so the generalisability of our results to other cohorts and milder CHD types needs to be determined. In addition, the observation of reduced foetal cerebral vascular resistance is a recognised feature of certain severe types of CHD, and although we adjusted for single-ventricle physiology, we recognise that this adjustment does not fully resolve or account for lesion-specific physiology. Disentangling foetal lesion-specific effects from maternal haemodynamic influences will become possible as more participants are recruited and a lesion-stratified analysis can be performed. Future work would also benefit from improved characterisation of the maternal foetal inferface, including uterine artery Doppler measures, to better assess maternal uteroplacental vascular resistance and its relationship to foetal cerebral haemodynamics in CHD. While the single centre study nature could minimise the external factors that could contribute to variation in maternal BP measurements, it requires replication to assess generalisability of the findings. BP itself only represents a snapshot of maternal cardiovascular function, and measurements were taken in a clinical setting at a single timepoint during pregnancy for each participant. Future studies would benefit from gathering longitudinal, ambulatory monitoring data, including more comprehensive measurement of maternal cardiac output. For Doppler and MRI, increasing sampling across pregnancy would clarify trimester-specific effects. We were insufficiently powered to examine the relationship between Doppler and maternal BP in control pregnancies, and a future study would benefit from a larger control group. Additionally, this observational study does not allow us to infer causality between maternal and foetal systems. To move towards mechanistic inference, a randomised control trial design would be needed. Overall, this study highlights the potential for a close interplay between maternal and foetal physiology in CHD, underscoring the need for further work to investigate maternal cardiovascular physiology as a potential regulator of foetal maturation in at-risk clinical groups.

## Contributors

SW, CKR, SM, and KI conceptualised the hypotheses and the methodological approach of the study. SW, CKR, and KI accessed and verified the underlying data. MRI data were collected by KI and CKR, image processing, quality control, and extraction of neuroimaging metrics were handled by SW and SJ, who implemented preprocessing pipelines and custom processing scripts, while SW conducted the statistical analysis, including group comparisons and outcome modelling. Interpretation of maternal–foetal interactions and discussion of their clinical significance was done by SM, CKR, PEG, LWH and SW. The final manuscript was read and approved by all authors.

## Data sharing statement

Image processing and analysis code for the manuscript is available via the centralised lab Github (https://github.com/FNNDSC). Anonymised data including MRI scans, metadata and derivative files used in this study will be available upon reasonable request with investigator support (caitlin.rollins@childrens.harvard.edu; kiho.im@childrens.harvard.edu), after signing a data sharing and usage agreement as required by the institution.

## Declaration of interests

S.U.M was supported by March of Dimes, American Heart Association. The other authors have no competing interests to disclose.
