# Supplementary material for: Linking maternal blood pressure with fetal cerebral haemodynamics and cortical growth in congenital heart disease
Source: eBioMedicine. 2026 Jul 6;130:106367. doi: 10.1016/j.ebiom.2026.106367 (PMC13351450; doi:10.1016/j.ebiom.2026.106367)
Supplement: Supplementary Figs. S1 and S2 and Tables S1–S9 [file mmc1.pdf]

Supplementary Material

Variable	Subcategory	Group	Mean (SD) / Count (%)	Test Statistics
**Pre-pregnancy Maternal BMI**		CHD (n = 84)	26.6 (5.16)	Welch t = -2.81, df = 163.85, p = 0.005, 95% CI (-3.51, -0.61)
		Control (n = 102)	24.5 (5.06)	
**Pre-pregnancy BMI Categories**	Underweight (<18.5)	CHD	2 (2.4)	
		Control	3 (2.9)	
	Normal (18.5–25)	CHD	31 (36.9)	
		Control	58 (56.9)	
	Overweight (25–30)	CHD	34 (40.5)	
		Control	30 (29.4)	
	Obesity I (30–35)	CHD	11 (13.1)	
		Control	9 (8.8)	
	Obesity II (35+)	CHD	6 (7.1)	
		Control	2 (2.0)	
**Maternal Age (years)**		CHD	31.9 (4.54)	Welch t = -2.39, df = 165.21, p = 0.48, 95% CI (-1.84, 0.87)
		Control	33.4 (4.12)	
**Gestational Age (GA) at 1st MRI (weeks)**		CHD	28.58 (2.95)	
		Control	27.21 (2.19)	
**GA at 2nd MRI (weeks)**		CHD	36.43 (2.28)	
		Control	35.71 (1.83)	
Time between 1st and 2nd MRI		CHD	8.33(3.25)	Student t = -0.41, df = 74, p = 0.685, 95% CI (-1.73, 1.15)
		Control	8.01(2.91)	
**GA at Echo (weeks)**		CHD	26.68 (3.90)	
		Control	22.23 (2.72)	
**GA at Maternal BP Measurement (weeks)**		CHD	27.23 (6.35)	
		Control	21.53 (7.85)	
Time between Echo & Maternal BP Measurement		CHD	9.05 (7.83)	Student t = 2.33, df = 160.29, p = 0.021, 95% CI (0.43, 5.28)
		Control	11.91 (7.87)	
Time between MRI & Maternal BP Measurement		CHD	8.98 (7.2)	Student t = -1.34, df = 243, p = 0.180, 95% CI (-3.18, 0.60)
		Control	10.26 (7.34)	

Supplementary Table 1. Comparison of Maternal Characteristics and Timing of Imaging and Blood Pressure Measurements Between CHD and Control Pregnancies. Summary of maternal demographics, gestational ages, and timing intervals for pregnancies affected by congenital heart disease (CHD) and controls. Continuous variables are presented as mean (standard deviation), and categorical variables as count (percentage). Between-group comparisons for continuous variables were performed using independent samples t-tests (Welch's t-test for unequal variances, Student's t-test for equal variances).

Group	Fetal CHD (n = 90)		No Fetal Anomaly (n = 60)	
Autoimmune Disorders (Examples: Lupus, Crohn's disease, psoriasis, Sjögren's, SSB antibodies)	5	5.56 %	2	3.33 %
Cardiac Disease	1 (Bicuspid Valve)	1.11 %	1 (Bicuspid Valve)	1. 67 %
Thyroid Disorders	Hypothyroidism: 4<br>(Treated = 3)<br>Hyperthyroidism: 1	4.44 %<br>(3.33 %)<br>1.11 %	Hypothyroidism: 8<br>(Treated = 5)<br>Hyperthyroidism: 0	13.33 %<br>(8.33 %)<br>0
Psychiatric Conditions	Anxiety: 23<br>Depression: 16<br>PTSD: 1<br>Bipolar: 1	25.56 %<br>17.78 %<br>1.11 %<br>1.11 %	Anxiety: 10<br>Depression: 8<br>PTSD: 1<br>Bipolar: 2	16.67 %<br>13.33 %<br>1.11 %<br>3.33 %
Reproductive Health Issues	PCOS: 12<br>Uterine Abnormalities: 6<br>Infertility: 5<br>History of Preterm Delivery: 6	13.33 %<br>6.67 %<br>5.56 %<br>6.67 %	PCOS: 3<br>Uterine Abnormalities: 5<br>Infertility: 3<br>History of Preterm Delivery: 2	5.00 %<br>8.33 %<br>5.00 %<br>3.33 %
Chronic Conditions	Diabetes: 4<br>Chronic Hypertension: 2<br>Kidney Disease: 3<br>Lung Disease: 4	4.44 %<br>2.22 %<br>3.33 %<br>4.44 %	Diabetes: 1<br>Chronic Hypertension: 1<br>Kidney Disease: 3<br>Lung Disease: 3	1.11 %<br>1.11 %<br>5.00 %<br>5.00 %
Neurological	Epilepsy: 1<br>Migraine: 4	1.11%<br>4.44%	Epilepsy: 1<br>Migraine: 8	1.11 %<br>13.33 %
**Pregnancy Related Health Concerns**				
Health Issues During Pregnancy	n = 29	32.22 %	n = 25	41.67 %
Gestational Diabetes	7	7.78 %	3	5.00 %
Pregnancy-Induced Hypertension	3	3.33 %	1	1.11 %
Infections During Pregnancy	UTIs: 8<br>Non-genital (e.g., colds, sinusitis): 11<br>Genital (e.g., HSV, yeast): 1	8.89 %<br><br>12.22 %<br>1.11 %	UTIs: 4<br>Non-genital (e.g., colds, sinusitis): 6<br>Genital (e.g., HSV, yeast): 1	6.67 %<br>10.00 %<br>1.11 %
Other Complications:	Antepartum Hemorrhage: 1<br>Preeclampsia/Eclampsia: 2<br>Maternal Pyrexia (fever): 6	1.11 %<br>2.22 %<br>6.67 %	Antepartum Hemorrhage: 2<br>Preeclampsia/Eclampsia: 2<br>Maternal Pyrexia (fever): 2	3.33 %<br>3.33 %<br>3.33 %
**Medications During Pregnancy**				
Prenatal Vitamins	82	91.11 %	47	78.33 %
Insulin	6	6.67 %	2	3.33 %
Thyroid Hormones (e.g., Levothyroxine)	7	7.78 %	5	8.33 %
Antibiotics	14	15.56 %	12	20.00 %
Progesterone	9	10.00 %	5	8.33 %
Anti-Nausea (e.g., Zofran, Diclegis)	11	12.22 %	8	13.33 %
Anti-Reflux	12	13.33%	11	18.33 %
Antidepressants	8	8.89 %	7	11.67 %
Steroids	9	10.00 %	4	6.67 %
Anti-Hypertensive	3	3.33 %	3	5.00 %
Aspirin	8	8.89 %	7	11.67 %
Anticoagulant (eg.,Waferin, Lovenox)	5	5.56 %	2	3.33 %

Supplementary Table 2. Maternal Medical History in Fetal CHD vs. No Fetal Anomaly (Control)

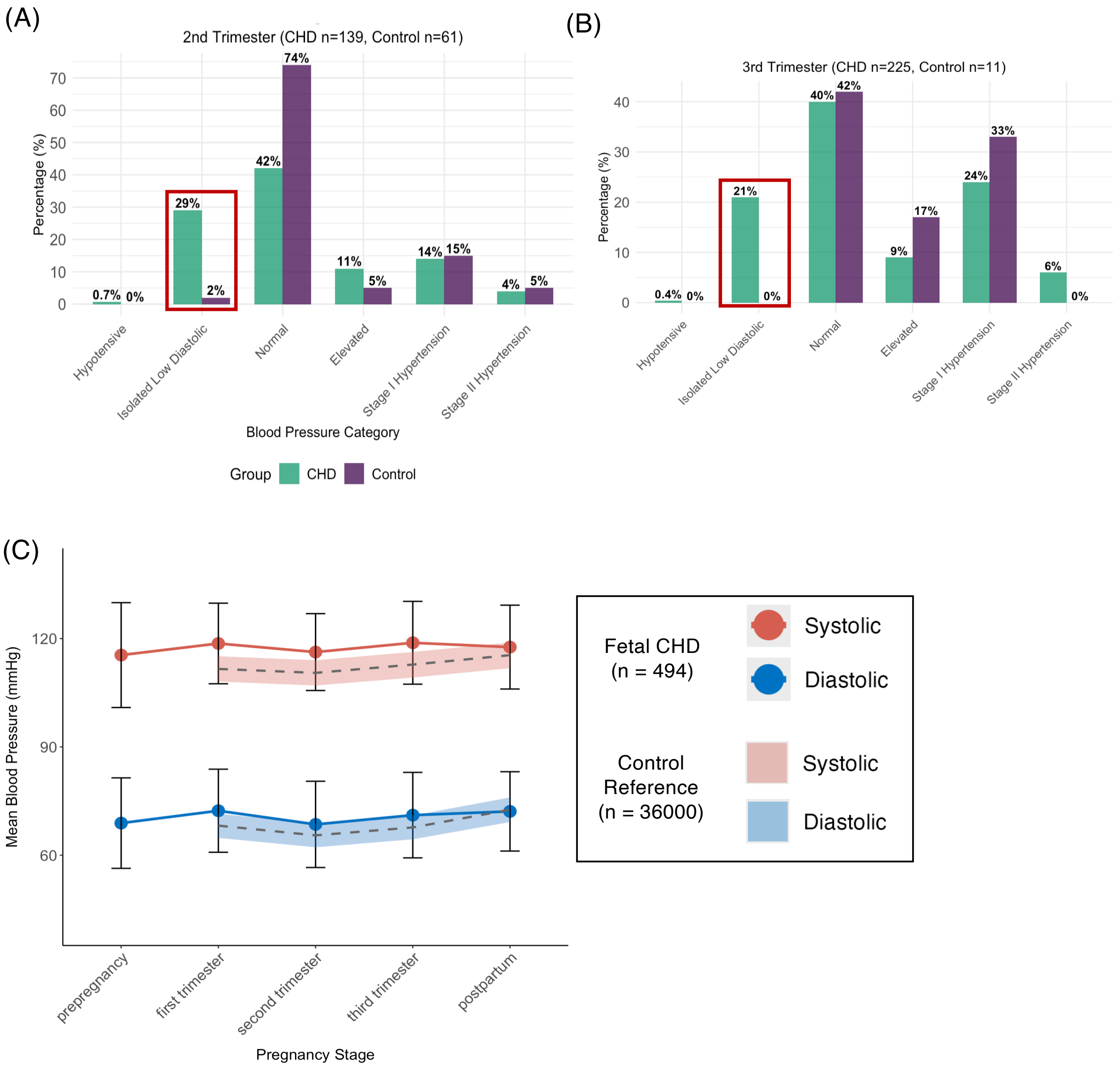

Supplementary Figure 1. Maternal blood pressure by pregnancy stage. (A) Second trimester maternal blood pressure distribution: CHD (green) vs control pregnancies (purple) (B) Third trimester maternal blood pressure distribution: CHD vs control pregnancies (C) Cross-sectional Maternal Blood Pressure trends in CHD pregnancies (n = 494) overlaid on a Reference Cohort (n = 36000) (data used with permission from Loerup et al., 2019) overlaid on a Control Reference Cohort (grey dotted line) (n = 36000, data used with permission from Loerup et al., 2019). Error bars represent the standard deviation.

Metric	CHD Median (IQR)	Control Median (IQR)	Non-parametric t-test (CHD vs. Control)	Hartigan's Dip Test p (CHD)	Hartigan's Dip Test p (Control)
**Systolic**	0.59 [±1.43]	0.45 [±1.14]	W = 17902, p = 0.18	D = 0.01, p = 0.75	D = 0.06, **p = 0.02**
**Diastolic**	0.44 [±2.63]	0.81 [±1.83]	W = 13618, **p = 0.01**	D = 0.04, **p = 0.001**	D = 0.04, p = 0.08
**MAP**	0.59 [±2.21]	0.71 [±1.61]	W = 14933, p = 0.20	D = 0.03, **p = 0.03**	D = 0.03, p = 0.72

Supplementary Table 3. Comparison of maternal blood pressure z-scores between Fetal CHD and Control (No fetal anomaly) groups. Values are presented as median [interquartile range]. Group differences were assessed using the Wilcoxon rank-sum test (reported as W statistic and p-value). Hartigan’s Dip Test was used to evaluate bimodality in the distribution of z-scores in each group. Significant p-values (<0.05) are shown in bold in the table.

	Cortical Surface Area (Z-score) ~ **maternal diastolic** + fetal age + fetal sex + maternal age + maternal bmi								Cortical Surface Area (Z-score) ~**maternal systolic** + fetal age + fetal sex + maternal age + maternal bmi							
	Left				Right				Left				Right			
**Brain Region**	**Beta**	**SE**	**FDR_P**	**Sig**	**Beta**	**SE**	**FDR_P**	**Sig**	**Beta**	**SE**	**FDR_P**	**Sig**	**Beta**	**SE**	**FDR_P**	**Sig**
**caudalmiddlefrontal**	-0.027	0.010	0.028	*	-0.023	0.008	0.014	*	0.009	0.012	0.787	ns	0.013	0.010	0.456	ns
**cingulatecortex**	-0.005	0.008	0.648		-0.004	0.009	0.743		0.006	0.011	0.787	ns	0.008	0.011	0.654	ns
**cuneus**	-0.012	0.008	0.217		0.005	0.008	0.632		0.009	0.010	0.747	ns	0.017	0.009	0.369	ns
**frontalpole**	-0.001	0.011	0.969		-0.012	0.008	0.196		-0.001	0.013	0.986	ns	0.005	0.010	0.699	ns
**fusiform**	-0.019	0.006	0.014	*	-0.013	0.008	0.155		-0.003	0.007	0.835	ns	0.006	0.010	0.695	ns
**inferiorparietal**	-0.029	0.010	0.028	*	-0.016	0.010	0.155		0.006	0.012	0.787	ns	-0.002	0.012	0.913	ns
**inferiortemporal**	0.000	0.008	0.983		-0.018	0.007	0.044	*	0.000	0.011	0.989	ns	-0.003	0.009	0.768	ns
**insula**	-0.016	0.007	0.062		-0.013	0.008	0.149		0.011	0.009	0.608	ns	0.014	0.009	0.369	ns
**isthmuscingulate**	0.017	0.012	0.241		0.003	0.008	0.756		-0.010	0.014	0.787	ns	-0.006	0.010	0.695	ns
**lateraloccipital**	-0.012	0.009	0.251		-0.028	0.009	0.014	*	0.007	0.011	0.787	ns	0.013	0.011	0.456	ns
**lateralorbitofrontal**	-0.023	0.009	0.028	*	-0.025	0.007	0.006	**	0.024	0.010	0.608	ns	0.014	0.009	0.369	ns
**lingual**	-0.024	0.009	0.028	*	-0.010	0.006	0.149		0.016	0.010	0.608	ns	0.007	0.007	0.548	ns
**medialorbitofrontal**	-0.009	0.008	0.357		-0.026	0.009	0.014	*	0.012	0.010	0.608	ns	0.018	0.011	0.369	ns
**middletemporal**	-0.006	0.010	0.648		-0.022	0.009	0.044	*	-0.009	0.012	0.787	ns	-0.003	0.010	0.861	ns
**paracentral**	-0.020	0.008	0.028	*	-0.019	0.010	0.097		0.012	0.009	0.608	ns	0.017	0.011	0.369	ns
**parahippocampal**	-0.025	0.010	0.028	*	-0.001	0.008	0.973		0.020	0.011	0.608	ns	0.017	0.009	0.369	ns
**parsopercularis**	-0.007	0.008	0.523		-0.003	0.006	0.668		-0.014	0.010	0.608	ns	-0.003	0.007	0.699	ns
**parsorbitalis**	-0.015	0.006	0.039	*	-0.019	0.010	0.095		-0.003	0.008	0.835	ns	0.001	0.012	0.913	ns
**parstriangularis**	-0.024	0.009	0.028	*	-0.022	0.009	0.044	*	-0.016	0.010	0.608	ns	-0.008	0.011	0.654	ns
**pericalcarine**	0.000	0.008	0.983		0.000	0.007	0.992		0.007	0.010	0.787	ns	0.013	0.008	0.369	ns
**postcentral**	-0.022	0.009	0.044	*	-0.033	0.009	0.005	**	0.007	0.011	0.787	ns	0.019	0.011	0.369	ns
**precentral**	-0.020	0.008	0.028	*	-0.031	0.008	0.005	**	0.014	0.010	0.608	ns	0.016	0.010	0.369	ns
**precuneus**	0.001	0.007	0.969		0.002	0.008	0.814		0.013	0.008	0.608	ns	0.007	0.010	0.654	ns
**rostralmiddlefrontal**	-0.038	0.010	0.007	**	-0.032	0.009	0.005	**	0.003	0.012	0.882	ns	0.012	0.010	0.456	ns
**superiorfrontal**	-0.014	0.010	0.241		-0.020	0.010	0.094		0.004	0.012	0.835	ns	0.010	0.012	0.654	ns
**superiorparietal**	-0.009	0.008	0.385		-0.014	0.008	0.138		0.011	0.011	0.747	ns	0.011	0.011	0.548	ns
**superiortemporal**	-0.025	0.008	0.022	*	-0.030	0.010	0.014	*	0.006	0.010	0.787	ns	0.017	0.011	0.369	ns
**supramarginal**	-0.017	0.008	0.085		-0.018	0.009	0.094		0.019	0.011	0.608	ns	0.016	0.011	0.369	ns
**temporalpole**	0.007	0.010	0.609		-0.005	0.005	0.395		0.006	0.012	0.787	ns	0.006	0.006	0.497	ns
**transversetemporal**	-0.023	0.007	0.014	*	-0.017	0.008	0.067		0.010	0.010	0.706	ns	0.019	0.009	0.369	ns

Supplementary Table 4. The effect of maternal diastolic BP (left) and systolic BP (right) on fetal cortical surface area in CHD

	Left Hemisphere			Right Hemisphere			Left Hemisphere			Right Hemisphere		
**Brain Region**	**Diastolic β (SE), 95% CI**	**FDR p**	**Sig**	**Diastolic β (SE), 95% CI**	**FDR p**	**Sig**	**Systolic β (SE), 95% CI**	**FDR p**	**Sig**	**Systolic β (SE), 95% CI**	**FDR p**	**Sig**
**caudalmiddlefrontal**	-0.027 (0.010), -0.047 to -0.007	0.028	*	-0.023 (0.008), -0.039 to -0.007	0.014	*	0.009 (0.012), -0.015 to 0.033	0.787	ns	0.013 (0.010), -0.007 to 0.033	0.456	ns
**fusiform**	-0.019 (0.006), -0.031 to -0.007	0.014	*	-0.013 (0.008), -0.029 to 0.003	0.155		-0.003 (0.007), -0.017 to 0.011	0.835	ns	0.006 (0.010), -0.014 to 0.026	0.695	ns
**inferiorparietal**	-0.029 (0.010), -0.049 to -0.009	0.028	*	-0.016 (0.010), -0.036 to 0.004	0.155		0.006 (0.012), -0.018 to 0.030	0.787	ns	-0.002 (0.012), -0.026 to 0.022	0.913	ns
**inferiortemporal**	0.000 (0.008), -0.016 to 0.016	0.983		-0.018 (0.007), -0.032 to -0.004	0.044	*	0.000 (0.011), -0.022 to 0.022	0.989	ns	-0.003 (0.009), -0.021 to 0.015	0.768	ns
**lateraloccipital**	-0.012 (0.009), -0.030 to 0.006	0.251		-0.028 (0.009), -0.046 to -0.010	0.014	*	0.007 (0.011), -0.015 to 0.029	0.787	ns	0.013 (0.011), -0.009 to 0.035	0.456	ns
**lateralorbitofrontal**	-0.023 (0.009), -0.041 to -0.005	0.028	*	-0.025 (0.007), -0.039 to -0.011	0.006	**	0.024 (0.010), 0.004 to 0.044	0.608	ns	0.014 (0.009), -0.004 to 0.032	0.369	ns
**lingual**	-0.024 (0.009), -0.042 to -0.006	0.028	*	-0.010 (0.006), -0.022 to 0.002	0.149		0.016 (0.010), -0.004 to 0.036	0.608	ns	0.007 (0.007), -0.007 to 0.021	0.548	ns
**medialorbitofrontal**	-0.009 (0.008), -0.025 to 0.007	0.357		-0.026 (0.009), -0.044 to -0.008	0.014	*	0.012 (0.010), -0.008 to 0.032	0.608	ns	0.018 (0.011), -0.004 to 0.040	0.369	ns
**middletemporal**	-0.006 (0.010), -0.026 to 0.014	0.648		-0.022 (0.009), -0.040 to -0.004	0.044	*	-0.009 (0.012), -0.033 to 0.015	0.787	ns	-0.003 (0.010), -0.023 to 0.017	0.861	ns
**paracentral**	-0.020 (0.008), -0.036 to -0.004	0.028	*	-0.019 (0.010), -0.039 to 0.001	0.097		0.012 (0.009), -0.006 to 0.030	0.608	ns	0.017 (0.011), -0.005 to 0.039	0.369	ns
**parahippocampal**	-0.025 (0.010), -0.045 to -0.005	0.028	*	-0.001 (0.008), -0.017 to 0.015	0.973		0.020 (0.011), -0.002 to 0.042	0.608	ns	0.017 (0.009), -0.001 to 0.035	0.369	ns
**parsorbitalis**	-0.015 (0.006), -0.027 to -0.003	0.039	*	-0.019 (0.010), -0.039 to 0.001	0.095		-0.003 (0.008), -0.019 to 0.013	0.835	ns	0.001 (0.012), -0.023 to 0.025	0.913	ns
**parstriangularis**	-0.024 (0.009), -0.042 to -0.006	0.028	*	-0.022 (0.009), -0.040 to -0.004	0.044	*	-0.016 (0.010), -0.036 to 0.004	0.608	ns	-0.008 (0.011), -0.030 to 0.014	0.654	ns
**postcentral**	-0.022 (0.009), -0.040 to -0.004	0.044	*	-0.033 (0.009), -0.051 to -0.015	0.005	**	0.007 (0.011), -0.015 to 0.029	0.787	ns	0.019 (0.011), -0.003 to 0.041	0.369	ns
**precentral**	-0.020 (0.008), -0.036 to -0.004	0.028	*	-0.031 (0.008), -0.047 to -0.015	0.005	**	0.014 (0.010), -0.006 to 0.034	0.608	ns	0.016 (0.010), -0.004 to 0.036	0.369	ns
**rostralmiddlefrontal**	-0.038 (0.010), -0.058 to -0.018	0.007	**	-0.032 (0.009), -0.050 to -0.014	0.005	**	0.003 (0.012), -0.021 to 0.027	0.882	ns	0.012 (0.010), -0.008 to 0.032	0.456	ns
**superiortemporal**	-0.025 (0.008), -0.041 to -0.009	0.022	*	-0.030 (0.010), -0.050 to -0.010	0.014	*	0.006 (0.010), -0.014 to 0.026	0.787	ns	0.017 (0.011), -0.005 to 0.039	0.369	ns
**transversetemporal**	-0.023 (0.007), -0.037 to -0.009	0.014	*	-0.017 (0.008), -0.033 to -0.001	0.067		0.010 (0.010), -0.010 to 0.030	0.706	ns	0.019 (0.009), 0.001 to 0.037	0.369	ns

Cortical Surface Area (Z-score) **~ maternal systolic + maternal diastolic** + fetal age + fetal sex + maternal age + maternal bmi											
		**Left Hemisphere**					**Right Hemisphere**				
**Cortical Region**	**Maternal Predictor**	**Beta**	**SE**	**P**	**FDR_P**	**Significant**	**Beta**	**SE**	**P**	**FDR_P**	**Significant**
**caudalmiddlefrontal**	diastolic	0.013	0.013	0.310	0.904	ns	0.004	0.013	0.740	0.954	ns
**caudalmiddlefrontal**	systolic	-0.002	0.011	0.873	0.898	ns	0.014	0.012	0.271	0.504	ns
**cingulatecortex**	diastolic	0.007	0.012	0.577	0.904	ns	-0.009	0.014	0.489	0.954	ns
**cingulatecortex**	systolic	0.004	0.011	0.702	0.853	ns	0.015	0.012	0.242	0.504	ns
**cuneus**	diastolic	0.001	0.011	0.938	0.955	ns	-0.005	0.011	0.622	0.954	ns
**cuneus**	systolic	0.005	0.010	0.624	0.814	ns	0.002	0.010	0.836	0.865	ns
**frontalpole**	diastolic	0.004	0.012	0.753	0.904	ns	0.006	0.013	0.627	0.954	ns
**frontalpole**	systolic	0.015	0.011	0.177	0.511	ns	0.023	0.012	0.054	0.448	ns
**fusiform**	diastolic	0.008	0.007	0.268	0.904	ns	-0.004	0.008	0.600	0.954	ns
**fusiform**	systolic	0.002	0.006	0.788	0.898	ns	0.007	0.008	0.355	0.561	ns
**inferiorparietal**	diastolic	-0.011	0.010	0.265	0.904	ns	0.005	0.013	0.672	0.954	ns
**inferiorparietal**	systolic	0.017	0.009	0.072	0.329	ns	0.012	0.011	0.286	0.504	ns
**inferiortemporal**	diastolic	0.012	0.011	0.303	0.904	ns	0.001	0.009	0.942	0.961	ns
**inferiortemporal**	systolic	-0.002	0.010	0.863	0.898	ns	0.007	0.008	0.439	0.598	ns
**insula**	diastolic	0.001	0.013	0.927	0.955	ns	-0.014	0.015	0.343	0.954	ns
**insula**	systolic	0.028	0.012	0.026	0.329	ns	0.027	0.013	0.050	0.448	ns
**isthmuscingulate**	diastolic	0.011	0.013	0.385	0.904	ns	-0.011	0.012	0.368	0.954	ns
**isthmuscingulate**	systolic	0.003	0.012	0.829	0.898	ns	0.022	0.011	0.048	0.448	ns
**lateraloccipital**	diastolic	-0.008	0.010	0.456	0.904	ns	0.006	0.009	0.507	0.954	ns
**lateraloccipital**	systolic	0.017	0.009	0.065	0.329	ns	0.004	0.009	0.623	0.747	ns
**lateralorbitofrontal**	diastolic	-0.013	0.012	0.287	0.904	ns	-0.003	0.012	0.804	0.954	ns
**lateralorbitofrontal**	systolic	0.020	0.011	0.082	0.329	ns	0.016	0.011	0.145	0.448	ns
**lingual**	diastolic	0.004	0.011	0.683	0.904	ns	0.003	0.009	0.737	0.954	ns
**lingual**	systolic	0.007	0.010	0.469	0.740	ns	0.007	0.009	0.404	0.598	ns
**medialorbitofrontal**	diastolic	-0.012	0.012	0.329	0.904	ns	-0.001	0.012	0.961	0.961	ns
**medialorbitofrontal**	systolic	0.020	0.011	0.088	0.329	ns	0.016	0.011	0.164	0.448	ns
**middletemporal**	diastolic	0.008	0.011	0.498	0.904	ns	0.002	0.008	0.827	0.954	ns
**middletemporal**	systolic	0.007	0.010	0.502	0.753	ns	0.012	0.007	0.120	0.448	ns
**paracentral**	diastolic	0.006	0.011	0.570	0.904	ns	0.010	0.013	0.451	0.954	ns
**paracentral**	systolic	0.006	0.010	0.534	0.763	ns	0.006	0.012	0.648	0.747	ns
**parahippocampal**	diastolic	-0.007	0.006	0.233	0.904	ns	-0.006	0.007	0.432	0.954	ns
**parahippocampal**	systolic	0.009	0.005	0.086	0.329	ns	0.005	0.007	0.434	0.598	ns
**parsopercularis**	diastolic	0.009	0.012	0.475	0.904	ns	0.002	0.010	0.813	0.954	ns
**parsopercularis**	systolic	0.009	0.011	0.394	0.739	ns	0.010	0.009	0.252	0.504	ns
**parsorbitalis**	diastolic	0.004	0.012	0.716	0.904	ns	0.001	0.012	0.911	0.961	ns
**parsorbitalis**	systolic	0.019	0.011	0.082	0.329	ns	0.005	0.011	0.638	0.747	ns
**parstriangularis**	diastolic	0.013	0.011	0.228	0.904	ns	0.009	0.010	0.375	0.954	ns
**parstriangularis**	systolic	0.005	0.010	0.619	0.814	ns	0.001	0.009	0.950	0.950	ns
**pericalcarine**	diastolic	-0.009	0.010	0.376	0.904	ns	0.004	0.009	0.683	0.954	ns
**pericalcarine**	systolic	0.001	0.009	0.898	0.898	ns	-0.002	0.008	0.795	0.852	ns
**postcentral**	diastolic	0.001	0.012	0.955	0.955	ns	-0.020	0.012	0.092	0.954	ns
**postcentral**	systolic	0.008	0.011	0.445	0.740	ns	0.004	0.011	0.731	0.812	ns
**precentral**	diastolic	-0.006	0.013	0.663	0.904	ns	-0.013	0.013	0.332	0.954	ns
**precentral**	systolic	0.013	0.012	0.274	0.586	ns	0.012	0.012	0.315	0.525	ns
**precuneus**	diastolic	-0.006	0.010	0.554	0.904	ns	-0.010	0.013	0.441	0.954	ns
**precuneus**	systolic	0.012	0.009	0.205	0.511	ns	0.017	0.012	0.140	0.448	ns
**rostralmiddlefrontal**	diastolic	-0.008	0.012	0.509	0.904	ns	0.005	0.012	0.663	0.954	ns
**rostralmiddlefrontal**	systolic	0.024	0.011	0.025	0.329	ns	0.014	0.011	0.208	0.504	ns
**superiorfrontal**	diastolic	0.005	0.014	0.737	0.904	ns	-0.002	0.014	0.898	0.961	ns
**superiorfrontal**	systolic	0.022	0.013	0.107	0.356	ns	0.020	0.013	0.129	0.448	ns
**superiorparietal**	diastolic	0.002	0.009	0.848	0.955	ns	-0.009	0.011	0.410	0.954	ns
**superiorparietal**	systolic	0.007	0.009	0.421	0.740	ns	0.014	0.010	0.161	0.448	ns
**superiortemporal**	diastolic	0.006	0.015	0.665	0.904	ns	0.006	0.012	0.601	0.954	ns
**superiortemporal**	systolic	0.017	0.014	0.223	0.515	ns	0.018	0.011	0.111	0.448	ns
**supramarginal**	diastolic	-0.001	0.011	0.909	0.955	ns	-0.015	0.010	0.155	0.954	ns
**supramarginal**	systolic	0.009	0.010	0.363	0.727	ns	0.014	0.009	0.134	0.448	ns
**temporalpole**	diastolic	-0.004	0.008	0.637	0.904	ns	-0.005	0.006	0.387	0.954	ns
**temporalpole**	systolic	0.003	0.008	0.711	0.853	ns	0.006	0.006	0.266	0.504	ns
**transversetemporal**	diastolic	0.004	0.011	0.725	0.904	ns	0.009	0.010	0.358	0.954	ns
**transversetemporal**	systolic	0.013	0.010	0.202	0.511	ns	0.005	0.009	0.602	0.747	ns

Supplementary Table 5. The effect of maternal BP on fetal cortical surface area in Controls

Doppler Measure (Z-score)	Predictor	β (SE)	95% CI	p value	Significant
**UA PI**	GA at echo	0.09 (0.06)	-0.023 to 0.212	.115	
	Diastolic BP	-0.003 (0.02)	-0.038 to 0.033	.886	
	Male sex	-1.44 (0.41)	-2.255 to -0.619	<.001	***
	Single ventricle	0.42 (0.51)	-0.584 to 1.433	.404	
	Head circumference Z	-0.35 (0.18)	-0.713 to 0.021	.064	
**UA RI**	GA at echo	0.04 (0.06)	-0.073 to 0.149	.498	
	Diastolic BP	-0.015 (0.02)	-0.047 to 0.016	.336	
	Male sex	-0.49 (0.37)	-1.224 to 0.248	.190	
	Single ventricle	0.31 (0.41)	-0.520 to 1.137	.460	
	Head circumference Z	-0.26 (0.16)	-0.580 to 0.069	.120	
**MCA PI**	GA at echo	0.12 (0.09)	-0.066 to 0.304	.204	
	Diastolic BP	0.09 (0.03)	0.033 to 0.143	.002	**
	Male sex	-1.35 (0.65)	-2.651 to -0.046	.043	*
	Single ventricle	-0.23 (0.80)	-1.833 to 1.370	.774	
	Head circumference Z	0.08 (0.30)	-0.513 to 0.676	.786	
**MCA RI**	GA at echo	-0.15 (0.11)	-0.360 to 0.065	.170	
	Diastolic BP	0.04 (0.03)	-0.016 to 0.106	.148	
	Male sex	-0.99 (0.72)	-2.440 to 0.450	.173	
	Single ventricle	0.26 (0.84)	-1.420 to 1.938	.759	
	Head circumference Z	-0.04 (0.33)	-0.697 to 0.611	.897	
**MCA PI**	GA at echo	0.08 (0.10)	-0.113 to 0.282	.397	
	Systolic BP	0.02 (0.03)	-0.038 to 0.085	.448	
	Male sex	-1.02 (0.69)	-2.389 to 0.346	.141	
	Single ventricle	-0.27 (0.86)	-1.978 to 1.446	.758	
	Head circumference Z	0.01 (0.32)	-0.621 to 0.640	.975	
**MCA RI**	GA at echo	-0.15 (0.11)	-0.368 to 0.072	.184	
	Systolic BP	0.01 (0.03)	-0.061 to 0.073	.856	
	Male sex	-0.84 (0.73)	-2.303 to 0.618	.252	
	Single ventricle	0.16 (0.85)	-1.542 to 1.872	.847	
	Head circumference Z	-0.05 (0.33)	-0.718 to 0.617	.879	
**CPR**	GA at echo	0.04 (0.07)	-0.095 to 0.170	.577	
	Diastolic BP	0.05 (0.02)	0.013 to 0.092	.009	**
	Male sex	0.36 (0.47)	-0.577 to 1.299	.446	
	Single ventricle	-0.54 (0.58)	-1.689 to 0.607	.351	
	Head circumference Z	0.23 (0.21)	-0.197 to 0.660	.285	
**CPR**	GA at echo	0.01 (0.07)	-0.124 to 0.152	.839	
	Systolic BP	0.02 (0.02)	-0.019 to 0.067	.275	
	Male sex	0.56 (0.48)	-0.399 to 1.521	.248	
	Single ventricle	-0.60 (0.60)	-1.799 to 0.600	.322	
	Head circumference Z	0.18 (0.22)	-0.261 to 0.626	.415	

Supplementary Table 6. Multivariate regression of predictors of fetal Doppler Z-scores. Regression coefficients (β), standard errors (SE), and p-values are shown for associations between clinical/demographic predictors and Doppler indices, including umbilical artery pulsatility index (UA PI), umbilical artery resistance index (UA RI), middle cerebral artery pulsatility index (MCA PI), middle cerebral artery resistance index (MCA RI), and cerebroplacental ratio (CPR). Predictors included in the model were GA at Echo, maternal blood pressure (systolic and diastolic), fetal sex, single ventricle physiology, and fetal head circumference Z-score. Significant associations are denoted as $p < .05$ (*) and $p < .01$ (**).

Pregnancies with fetal CHD	Placental Pathology Follow-up (n)	Value<br>74
Placental Biometry	Placenta Weight (g), median (IQR)	468.5 (403.3 - 536.8)
	<10th Percentile, n (%)	6 (8%)
	>90th Percentile, n (%)	7 (9%)
	Placenta Length (cm), median (IQR)	20.0 (18.0 - 21.3)
	Placenta Width (cm), median (IQR)	17.5 (16.0 - 19.0)
	Mural Thickness (cm), median (IQR)	3 (2.7 - 3.3)
**Umbilical Cord**	Cord Diameter (cm), median (IQR)	1.4 (1.2 - 1.7)
	Single Umbilical Artery (n)(%)	4 (5%)
Cord Insertion (Normal)	Paracentral (n)(%)	55 (74%)
	Central (n)(%)	9 (12%)
Cord Insertion (Abnormal)	Marginal (n)(%)	4 (5%)
	Velamentous (n)(%)	2 (3%)
	Membranous (n)(%)	4 (5%)
**Maternal Vascular Malperfusion (MVM)**	MVM Present (n)(%)	23 (31%)
	Placental Infarct (n)(%)	12 (16%)
	Retroplacental Hemorrhage (n)(%)	5 (7%)
	Distal Villous Hypoplasia	5 (7%)
	Accelerated Villous Maturation	1 (1%)
	Decidual Vasculopathy	4 (5%)
	Syncytial Knots (n)(%)	4 (5%)
**Inflammation - Maternal Response**	Placental Inflammation Present (n)(%)	24 (32%)
	Inflammation - Chorion	5 (7%)
	Inflammation - Amnion	4 (5%)
	Inflammation - Subchorion	13 (18%)
	Inflammation - Chorionic Vessels	6 (8%)
	Inflammation - Cord	2 (3%)
	Inflammation - Umbilical Vein	3 (4%)
	Inflammation - Umbilical Artery	1 (1%)
Maternal Inflammation	Stage I	14 (19%)
	Stage II	4 (5%)
**Chronic Inflammatory Lesions**	Villitis of Unknown Etiology (VUE)	8 (11%)
**Fetal Vascular Malperfusion (FVM)**	FVM Present (n)(%)	8 (11%)
	Thrombosis	4 (5%)
	Avascular Villi	4 (5%)
	Karyorrhexis	1 (1%)
Other Vascular Findings	Delayed Villous Maturation (DVM)	3 (4%)
	Chorangiosis	5 (7%)
Other Pathology	Meconium-Associated Changes	6 (8%)
	Thrombohematoma	10 (13%)
Summary	Placental Pathology Severity Score	

Supplementary Table 7. Placental Pathology in pregnancies with Fetal CHD Reported According to the Amsterdam Criteria.

Maternal BP ~ Maternal Vascular Malperfusion + Prepregnancy BMI + Maternal Age + Pregnancy Stage						
**BP**	**Predictor**	**df**	**Sum sq**	**Mean sq**	**f value**	**p value**
**Systolic**	MVM	1	0.560	0.560	0.005	0.941
	Prepregnancy BMI	1	107.298	107.298	1.043	0.312
	Maternal Age	1	0.649	0.649	0.006	0.937
	Pregnancy Stage	4	526.048	131.512	1.278	0.291
**Diastolic**	MVM	1	8.367	8.367	0.077	0.783
	PrepregnancyBMI	1	173.757	173.757	1.594	0.213
	Maternal Age	1	110.103	110.103	1.010	0.320
	Pregnancy Stage	4	426.373	106.593	0.978	0.428
**MAP**	MVM	1	2.819	2.819	0.035	0.853
	Prepregnancy BMI	1	149.833	149.833	1.855	0.179
	Maternal Age	1	45.249	45.249	0.560	0.458
	Pregnancy Stage	4	423.179	105.795	1.309	0.279

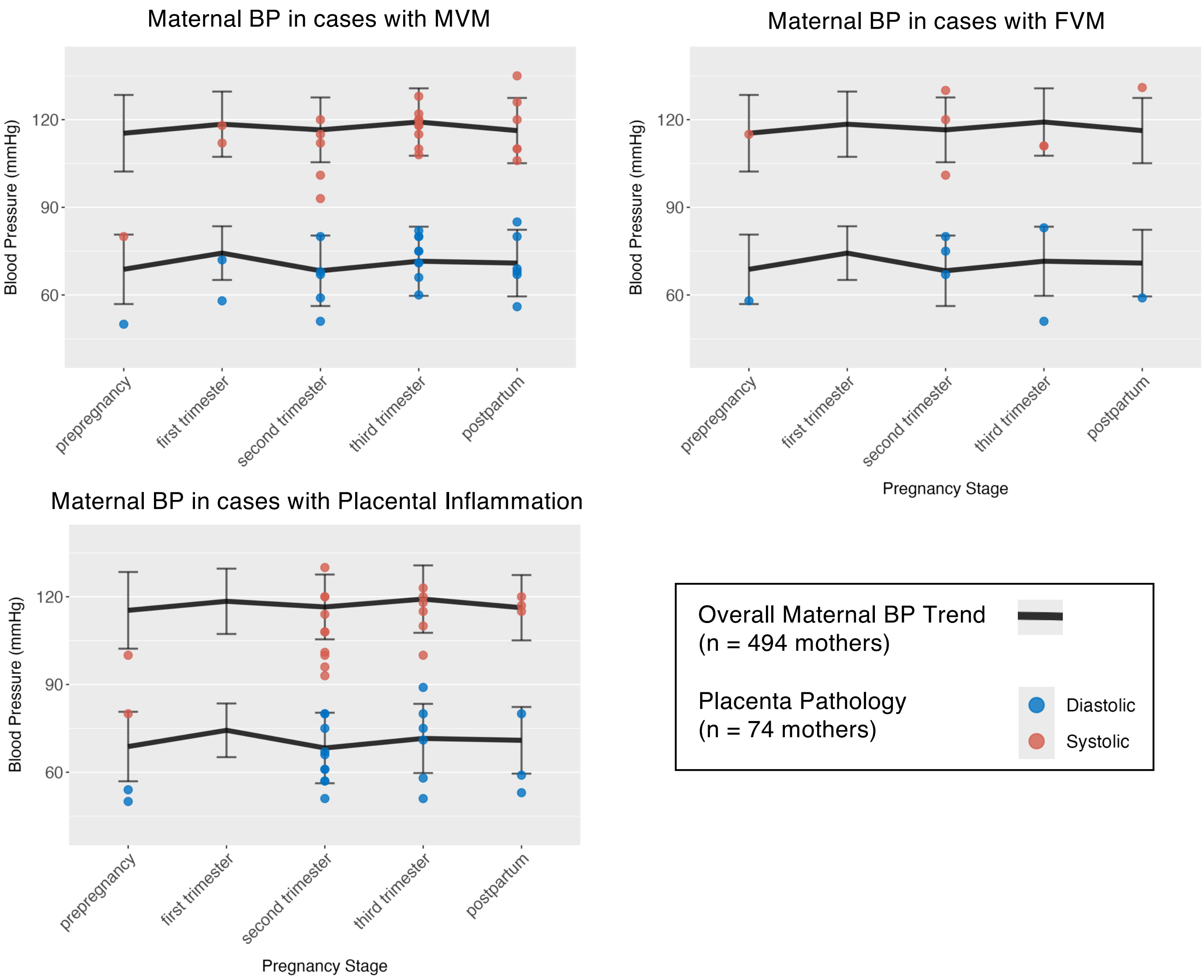

Supplementary Figure 2. Maternal blood pressure across pregnancy in fetal CHD showed no significant associations with placental predictors.

Analysis of variance results for the association between maternal vascular malperfusion (MVM), pre-pregnancy body mass index (BMI), maternal age, and pregnancy stage with maternal systolic blood pressure, diastolic blood pressure, and mean arterial pressure (MAP) in pregnancies affected by fetal congenital heart disease (CHD). Shown are degrees of freedom (df), sums of squares (Sum sq), mean squares (Mean sq), F values, and *p* values for each predictor. None of the predictors were significantly associated with maternal blood pressure measures. Corresponding plots display longitudinal maternal blood pressure trends across gestation with cases of placental pathology (MVM) overlaid on reference lines of mean maternal blood pressure, illustrating the lack of consistent deviation in MVM cases compared to the overall trajectory.

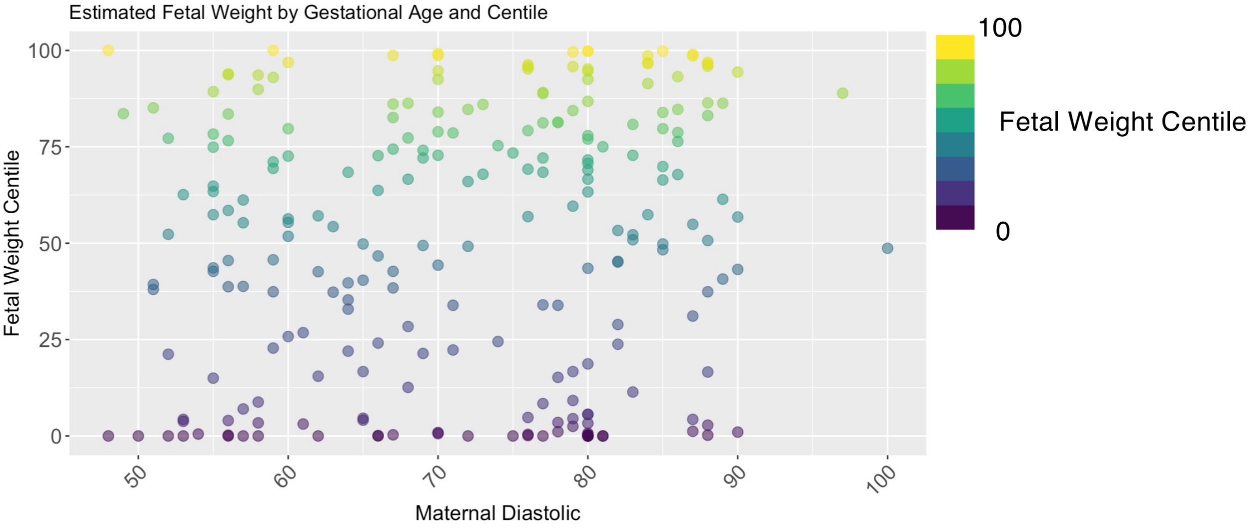

Fetal Weight Centile ~ Maternal BP + Fetal Gestational Age				
	**Estimate**	**SE**	**t**	**p**
**Diastolic**	0.33	0.19	1.72	0.09
**Systolic**	0.14	0.21	0.68	0.50

Supplementary Table 8. Maternal blood pressure is not a significant predictor of fetal weight in fetuses with moderate to severe CHD

Infant Birthweight Z-score						
**Group**	*n (Infants)*	*mean (sd)*	*min*	*max*	*CHD vs. Control (Welch Two Sample t-test)*	
**Control**	87	0.61 ± 1.12	-1.76	2.54	t = -3.0, df = 181.73, p = 0.003	95% CI (-0.73 -0.15)
**CHD (all)**	98	0.23 ± 1.04	-3.20	3.26		
**CHD (TGA)**	24	0.25 ± 1.14	-1.87	3.26		
**CHD (exc. TGA)**	74	0.27 ± 1.01	-3.2	2.56	t = -2.865, df = 151.02, p = 0.005	95% CI (-0.75 -0.14)

Infant Birthweight Z-score ~ Maternal Blood Pressure (during pregnancy) + Gestational Age at BP measurement			
**Statistic**	**Systolic BP**	**Diastolic BP**	**MAP**
**Residuals**			
Min	-3.23	-3.42	-3.4
1Q	-0.74	-0.75	-0.77
Median	0.002	0.035	0.032
3Q	0.51	0.59	0.56
Max	2.96	3.03	2.97
**Model Fit**			
Residual Std Error	0.99 (141 df)	1.01 (141 df)	0.99 (141 df)
Multiple R^2	0.013	0.0005	0.0034
Adjusted R^2	0.006	-0.006	-0.004
F-statistic	1.91 (1, 141 df)	0.07 (1, 141 df)	0.43 (1, 141 df)
p-value	0.17	0.79	0.49

Supplementary Table 9. Maternal blood pressure is not a significant predictor of birth weight in fetuses with moderate to severe CHD
